# Levetiracetam versus phenytoin/fosphenytoin for second-line treatment of children with convulsive status epilepticus: an up-to-date meta-analysis and systematic review of randomized controlled trials

**DOI:** 10.3389/fneur.2025.1580329

**Published:** 2025-05-29

**Authors:** Linping Jin, Zhiping Jin, Zhijiang Wang

**Affiliations:** Department of Pediatric, Tianxiang Medical Oriental Hospital, Yiwu, China

**Keywords:** convulsive status epilepticus, phenytoin, fosphenytoin, levetiracetam, seizure, review, meta-analysis

## Abstract

**Objective:**

To compare the efficacy and safety of levetiracetam versus phenytoin/fosphenytoin as second-line treatments in children with convulsive status epilepticus (CSE).

**Methods:**

A systematic search identified randomized controlled trials comparing levetiracetam with phenytoin/fosphenytoin to treat CSE in children. Fourteen studies involving 2,197 patients were included in the meta-analysis.

**Results:**

No significant difference was found between the two treatments regarding seizure cessation (odds ratio (OR): 1.18, 95% confidence interval (CI): 0.94–1.48; *p* = 0.16) or time to clinical seizure termination (mean difference: −0.10, 95% CI: −0.61 to 0.40; *p* = 0.69). However, levetiracetam was associated with significantly fewer seizure recurrences (OR: 0.60, 95% CI: 0.43–0.84; *p* = 0.003) and adverse events (OR: 0.59, 95% CI: 0.37–0.94; *p* = 0.03) compared with phenytoin/fosphenytoin. No significant differences were observed in the need for mechanical ventilation, intensive care unit admission, or hospital length of stay.

**Conclusion:**

Levetiracetam is as effective as phenytoin/fosphenytoin to control seizures in children with CSE and is associated with fewer seizure recurrences and adverse events.

## Background

Convulsive status epilepticus (CSE) is a serious neurological emergency in children that increases mortality and morbidity risks and has an incidence of 10–60 per 100,000 people. CSE requires prompt treatment and termination to obtain satisfactory outcomes (1, 2). Benzodiazepines are the first-line anticonvulsants for CSE; however, 35–60% of patients with CSE do not respond to this treatment (3, 4). Several conventional agents have been proposed as second-line treatment for children with CSE, including phenytoin and fosphenytoin (5); however, these drugs are associated with several adverse events. Levetiracetam was approved in 2006 for children and is a new anti-seizure medication. Recently, several studies demonstrated the safety and efficacy of levetiracetam when used to treat CSE (6, 7). Although levetiracetam has potent antiepileptic effects, there are potential side effects, including behavioral changes and sleep disturbances (8, 9). Various studies have compared phenytoin/fosphenytoin and levetiracetam in terms of safety and efficacy for second-line treatment of children with CSE; however, there is no clear evidence that confirms that one drug is superior to the other. We performed an up-to-date meta-analysis and systematic review of randomized controlled trials (RCTs) to compare the effectiveness of levetiracetam and phenytoin/fosphenytoin to treat pediatric cases of CSE.

## Methods

This meta-analysis was performed in accordance with the Preferred Reporting Items for Systematic Reviews and Meta-Analysis (PRISMA) guidelines (10). Institutional Review Board approval was not applicable for this study.

### Search strategy and selection criteria

Two authors independently performed a thorough electronic search of literature published before 1 January 2025 to identify original RCTs comparing levetiracetam and phenytoin/fosphenytoin treatment for children with CSE. We comprehensively searched the online databases of PubMed, Embase, Web of Science, the Cochrane Central Register of Controlled Trials (CENTRAL), and ClinicalTrials.gov. The English search terms included but were not limited to the following: “levetiracetam,” “phenytoin,” “fosphenytoin,” “epilepsy,” “seizures,” “convulsive status epilepticus,” and “antiepileptic.” The search was restricted to human subjects and English-language articles. We also manually reviewed the references of the articles identified in the initial search. Full-text articles and abstracts were included in this study. Retrospective studies, single-arm studies, review articles, case reports, editorials, and letters to the editor were excluded, as were duplicate publications by the same author or agency and studies with insufficient data for the outcome measures.

### Outcome measures and data extraction

The primary outcome of this study was seizure cessation within 24 h. In most included studies, seizure cessation was defined based on clinical observation alone, while a few studies incorporated EEG-confirmed seizure cessation. Given this variability, we acknowledge this as a limitation and emphasize the need for standardized criteria in future studies. Secondary outcome measures were time to seizure recurrence within 24 h, termination of clinical seizure, adverse events, requirement for mechanical ventilation, intensive care unit admission, and hospital stays. Three authors independently extracted the following data from the included studies: authors, publication year, sample size, patient age, and etiology. Conflicts regarding data abstraction were resolved by consensus and by referring to the original article. EndNote version X8 (Thomson Reuters, Toronto, ON, Canada) was used to remove duplicate studies.

### Assessment of methodological quality and risk of bias

Two authors independently evaluated the quality of the studies. The Cochrane collaboration tool (Center for Evidence-Based Medicine Odense and Cochrane Denmark, Denmark) was used to assess the quality of the RCTs by evaluating methods of randomization and allocation concealment, performance, and detection of bias.

### Statistical analysis

All statistical analyses were performed using Review Manager version 5.3 software (Cochrane Informatics and Knowledge Management Department, Nordic Cochrane Center, Copenhagen, Denmark). Odds ratios (ORs) with 95% confidence intervals (CIs) were used to describe dichotomous outcomes. Publication bias was evaluated by the *χ*^2^ test and funnel plots. The *I*^2^ test and *p*-values were used to test heterogeneity, which was considered significant with *I*^2^ > 50% and *p* < 0.05. A fixed-effects model was used when heterogeneity was not significant; otherwise, a random-effects model was used. A two-tailed *p*-value < 0.05 was considered statistically significant. We also assessed the potential for publication bias through a visual inspection of funnel plot asymmetry.

## Results

### Study selection and trial characteristics

**Figure 1:**
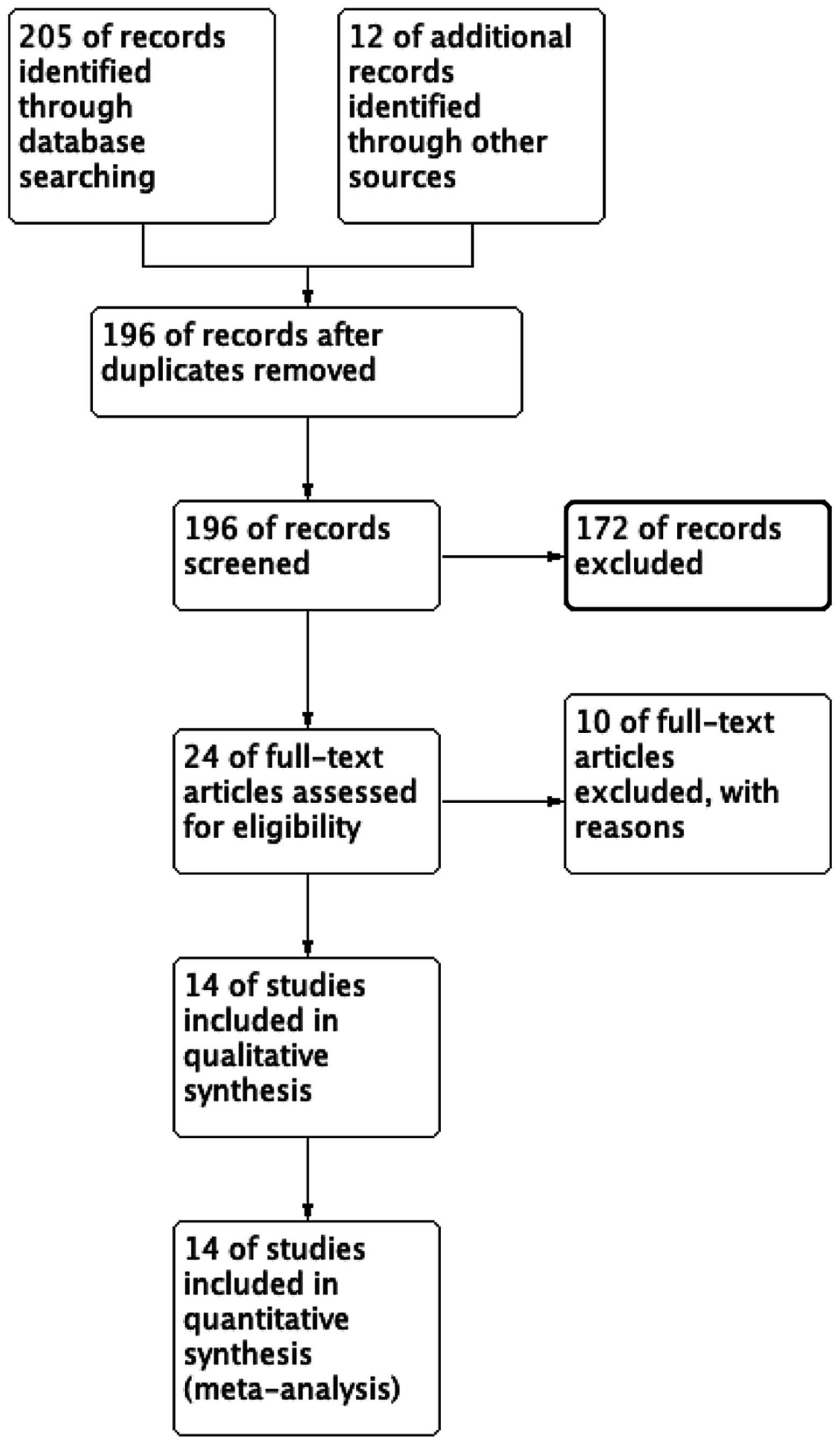
Study flowchart for patient selection.

The search identified 217 articles, 21 of which were removed as duplicates; a further 172 were excluded after title and abstract review. Ten additional studies were excluded after applying the exclusion criteria. In total, 14 studies were included in the final meta-analysis (9, 11–23). The selection process and flow chart are summarized in Figure 1.

**Figure 2:**
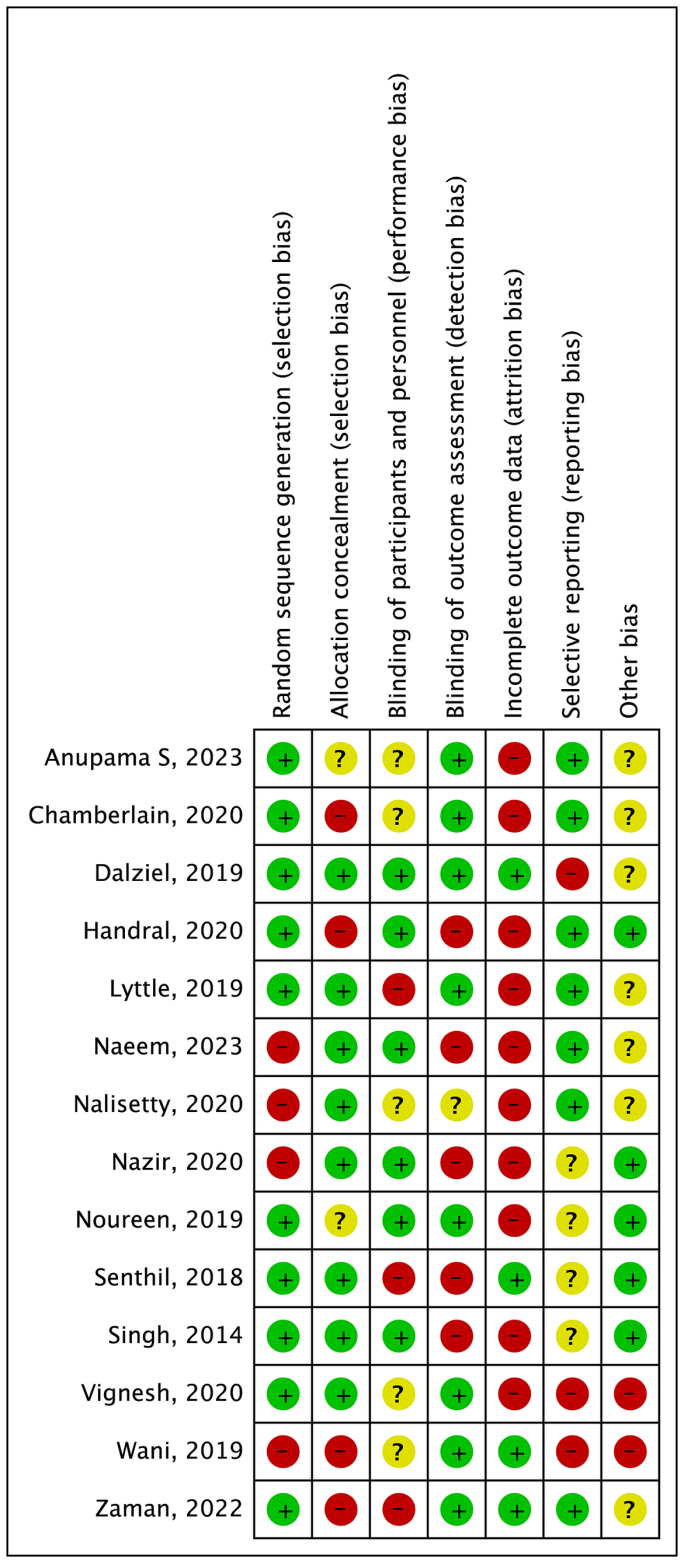
Consensus risk of bias assessment of the included studies. Green, low risk; yellow, unclear; red, high risk.

The 14 studies included RCTs involving 2,197 pediatric patients aged between 3 months and 16 years, who were enrolled into two groups (1,117 participants in the levetiracetam group and 1,080 participants in the phenytoin/fosphenytoin group). Among the 14 studies, 8 were performed in India, 3 in Pakistan, 1 in New Zealand, 1 in the USA, and 1 in the UK. All studies were published recently (2014–2023). The dose of levetiracetam varied from 20 to 60 mg/kg, while phenytoin/fosphenytoin was used at 20–30 mg/kg. The risk of bias is shown in Figure 2, and the characteristics of the included studies are shown in Table 1.

**Table 1:** Characteristics of included studies.

Study Year	Country	Study period	Sample, *n* (L/P)	Age, y	Gender (M/F)	Etiology
Anupama, et al. (21)	India	August 2016 September 2017	50/50	L: 26 (13.8–84) P: 30 (13–104)	L:33/17 P:26/24	NA
Chamberlain et al. (19)	USA	Nov 3, 2015 Dec 29, 2018	85/71	NA	124/101	Unprovoked, Febrile illness, Other
Dalziel et al (9)	New Zealand	March 19, 2015 Nov 29, 2017	119/114	L: 3.8 (3.8) P: 4.0 (3.9)	L:59/60 P:53/61	NA
Handral et al (20)	India	November 2013 June 2015	58/58	L: 3.09 (2.98) P: 3.77 (3.79)	L:32/26 P:36/22	Acute symptomatic, Remote symptomati, Febrile status, Idiopathic, Cryptogenic
Lyttle et al. (18)	UK	July 17, 2015 April 7, 2018	152/134	L: 2.7 (1.3–5.9) P: 2.7 (1.6–5.6)	L:75/77 P:72/62	Febrile convulsion, Seizure (pre-existing epilepsy), First afebrile seizure, CNS infection, Intracranial vascular event (bleed or stroke), Traumatic brain injury, Substance misuse, Indeterminate, Other
Naeem et al. (12)	Pakistan	November 2020 May 2021	67/67	NA	L:45/22 P:48/19	Meningitis/encephalitis, Febrile seizures, Epilepsy, Cerebral palsy and epilepsy, Neurodegenerative disorders and epilepsy
Nalisetty et al. (17)	India	June 2014 Dec 2015	32/29	L: 29.4 (31.2) P: 32.9 (37.2)	L:16/16 P:16/13	Febrile seizure, Encephalitis, Unprovoked seizure, Camphor poisoning, Fever provoked seizure
Nazir et al. (20)	India	2012 2014	50/50	L: 4.98 (4.14) P: 5.17 (3.71)	L:36/14 P:35/15	Idiopathic, Congenital/perinatal, Febrile status, Infections, Vascular
Noureen et al. (13)	Pakistan	January 2014 June 2018	300/300	L: 3.52 (0.24) P: 3.46(0.22)	L:216/84 P:190/110	Meningitis/encephalitis, Febrile seizures, Epilepsy, Cerebral palsy and epilepsy, Neurodegenerative disorders and epilepsy
Senthil et al. (23)	India	January 2017 June 2017	25/25	L: 2.28 (2.19) P: 3.34 (3.36)	L:18/7 P:16/9	Cryptogenic, Acute CNS infection, Febrile seizures, Seizure disorder (non-compliance), Syndromic association, Hypoglycemia, Thulasi oil ingestion, Seizure disorder (break through seizures), Sepsis, Camphor ingestion, Post meningo-encephalitic sequelae
Singh et al. (16)	India	November 2012 April 2014	50/50	L: 7.35 (2.20) P: 7.03 (2.85)	L:32/18 P:26/24	NA
Vignesh et al. (14)	India	June, 2016 December, 2018	32/35	L: 58 (50) P: 44 (43)	L:18/14 P:19/16	Acute, Remote, Acute on remote, Febrile status, Epileptics, Unknown
Wani et al. (15)	India	NA	52/52	L: 3.39 (3.32) P: 4.80 (4.11)	L:32/20 P:34/18	NA
Zaman et al. (11)	Pakistan	Jan-Dec 2020	45/45	L: 8.31 (4.62) P: 8.88 (4.14)	L:21/24 P:25/20	NA

### Primary outcomes

#### Seizure cessation

**Figure 3:**
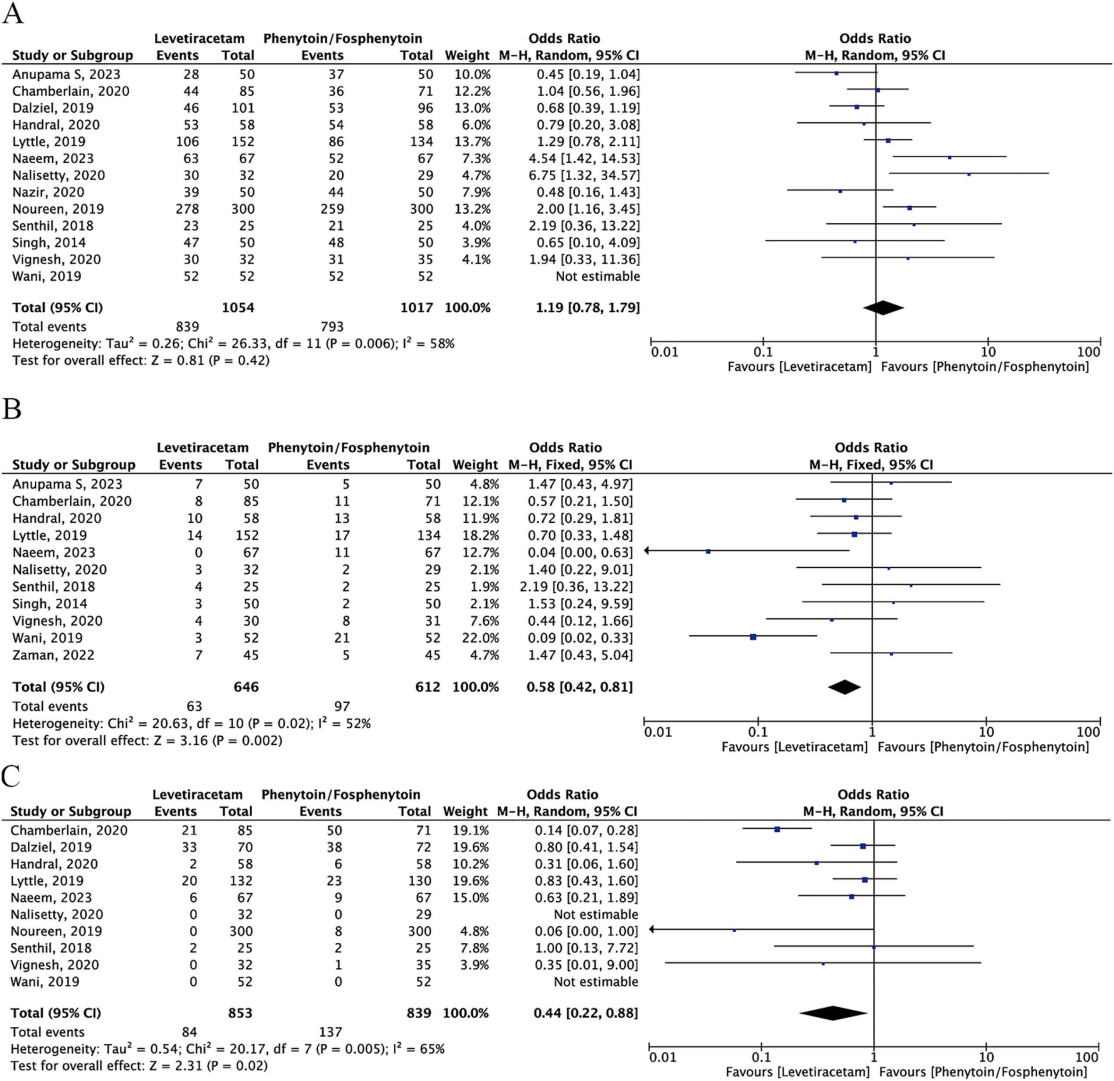
Forest plot of the meta-analysis comparing levetiracetam and phenytoin/fosphenytoin. **(A)** Seizure cessation; **(B)** Seizure recurrence; **(C)** Adverse events.

Data for seizure cessation were reported in all included trials, namely 839/1054 patients in the levetiracetam group and 793/1017 patients in the phenytoin/fosphenytoin group. There were no significant differences between the levetiracetam and phenytoin/fosphenytoin groups (OR: 1.19; 95% CI: 0.78–1.79), with *I*^2^ = 58% (Figure 3A).

#### Seizure recurrence

Eleven studies provided data for seizure recurrence for 63/646 patients in the levetiracetam group and 94/612 patients in the phenytoin/fosphenytoin group. Levetiracetam appeared to be associated with lower seizure recurrence rates compared with phenytoin/fosphenytoin (OR: 0.58; 95% CI: 0.42–0.81), with *I*^2^ = 52% (Figure 3B).

#### Adverse events

Ten studies reported 84/853 and 137/839 adverse events in the levetiracetam and phenytoin/fosphenytoin groups, respectively. Analysis showed that the levetiracetam group experienced fewer adverse events (OR: 0.59; 95% CI: 0.37–0.94), with *I*^2^ = 61% (Figure 3C).

#### Mechanical ventilation

**Figure 4:**
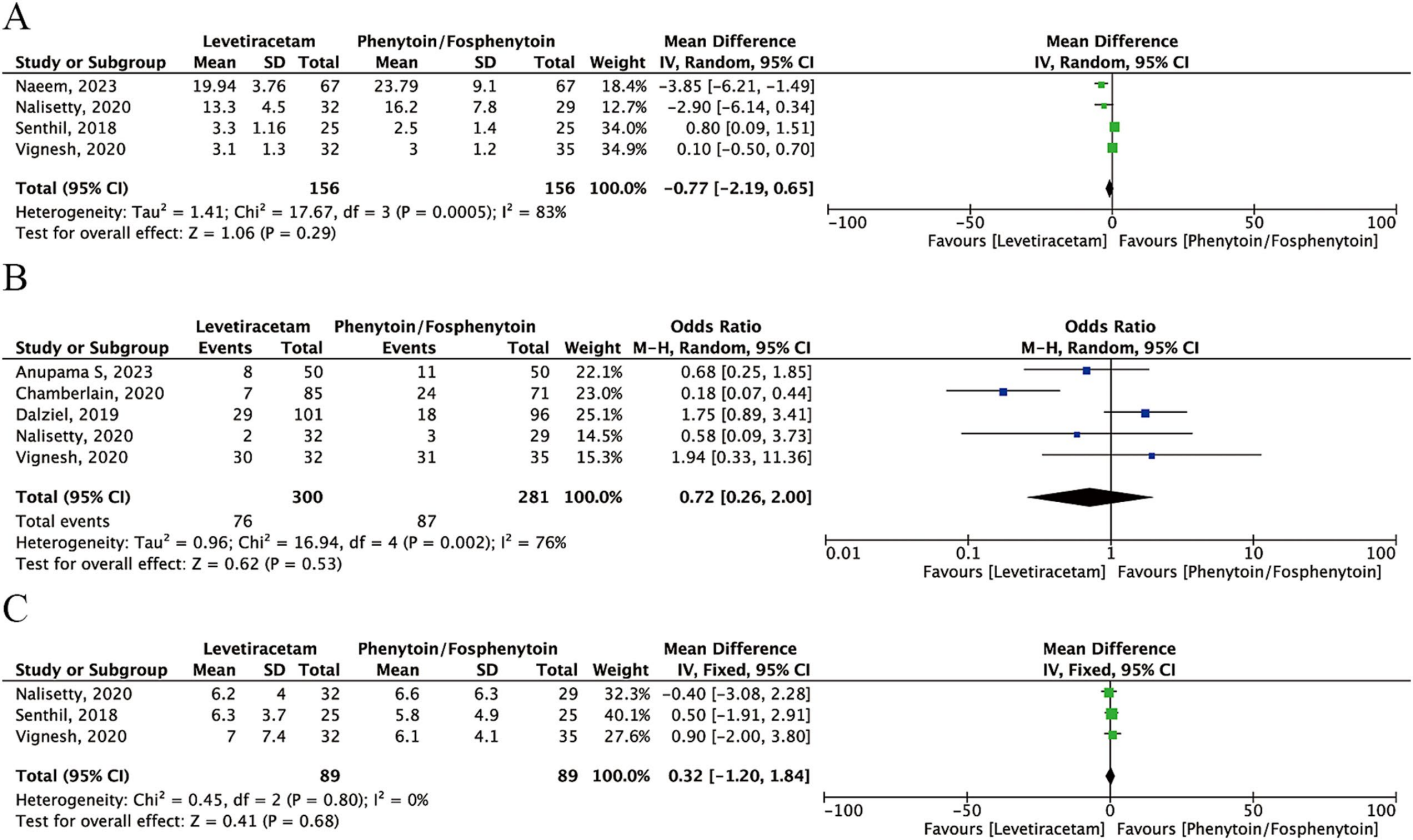
Forest plot of the meta-analysis comparing levetiracetam and phenytoin/fosphenytoin. **(A)** Mechanical ventilation; **(B)** Intensive care unit admission; **(C)** Hospital stays.

Five studies reported the number of patients that required mechanical ventilation. Analysis revealed no significant difference between the levetiracetam and phenytoin/fosphenytoin groups (OR: 0.72; 95% CI: 0.26–2.00), with *I*^2^ = 76% (Figure 4A).

#### Intensive care unit admission

Six studies reported 222/469 patients in the levetiracetam group and 192/432 in the phenytoin/fosphenytoin group who required intensive care unit admission, with no significant difference between the groups (OR: 1.09; 95% CI: 0.68–1.76), with *I*^2^ = 39% (Figure 4B).

#### Hospital stays

Three studies reported data on hospital stays. The analysis revealed no significant difference between the levetiracetam and phenytoin/fosphenytoin groups (mean difference: 0.32, 95% CI: −1.20–1.84), with *I*^2^ = 0% (Figure 4C).

### Sensitivity analysis and publication bias

The influence of a single study on the overall meta-analysis estimate was investigated by omitting one study at a time. The omission of any study resulted in no significant difference, indicating that our results were statistically reliable. Most graphical funnel plots of the parameters were symmetrical.

## Discussion

In the present study, we compared the efficacy and safety of levetiracetam and phenytoin/fosphenytoin for second-line treatment of children with CSE. To our knowledge, this is the largest and most recent meta-analysis of RCTs on this topic to date. Our study showed that levetiracetam has comparable efficacy outcomes and is associated with fewer adverse events compared with phenytoin/fosphenytoin.

For children with CSE, numerous guidelines suggest benzodiazepines as the first-line anticonvulsant treatment (5). Several RCTs have compared levetiracetam and phenytoin/fosphenytoin for CSE treatment (9, 21, 24). A recent RCT showed that levetiracetam was comparable to fosphenytoin as a second-line medication for the management of CSE (21). Similar results have been confirmed in several studies (15, 18); however, this conclusion was not corroborated by others. The ConSEPT trial, an open-label, multicenter RCT, showed that levetiracetam was not superior to phenytoin (9). A different meta-analysis included retrospective studies and RCTs and demonstrated that levetiracetam was superior to phenytoin in children with CSE (1). Another meta-analysis included nine studies with a total of 1732 patients, and showed no significant difference between the two drugs in terms of seizure cessation (25). Compared with previous studies, the strength of the present study lies in its inclusion of the latest RCTs. The most recent study was published in 2023, while others were published within the last decade. Our study showed that levetiracetam was comparable with phenytoin/fosphenytoin; however, CSE remains a complex disease state with diverse presentations and underlying causes, and previous antiepileptic drug usage can affect treatment results. The included RCTs exhibited considerable variability in the definition of seizures and seizure recurrence. In future research, more high-quality, large-sample-size RCTs are needed.

Several adverse events have been reported with phenytoin/fosphenytoin use, including cardiac reactions, arrhythmia, hypotension, and skin manifestations (26). The adverse events of levetiracetam include behavioral changes, sleep disturbances, diplopia, and gastrointestinal manifestations, such as nausea. In our study, 10 RCTs reported data on adverse events, with 84/853 in the levetiracetam group and 137/839 in the phenytoin/fosphenytoin group, indicating that levetiracetam treatment led to fewer adverse events compared with phenytoin/fosphenytoin. However, some studies did not report adverse events, which could have led to selection bias. Additionally, the definition of adverse events varied between the RCTs. Although our study primarily focused on pediatric patients, we acknowledge that the adverse effects of phenytoin may be influenced by patient age and underlying conditions, such as infections. To address this, we have expanded the discussion to explore how these factors might contribute to the observed variations in adverse event rates. Additionally, we recommend that future studies stratify adverse effects based on patient age and comorbid conditions to provide a more comprehensive understanding of these potential interactions.

Although this study included the latest and largest known number of RCT studies, there were several limitations. Firstly, the limited number of studies for some secondary outcomes reduces the statistical power and generalizability of our findings. This limitation may have led to less precise effect estimates, and caution should be exercised when interpreting these results. Future large-scale, multicenter randomized controlled trials (RCTs) with adequate power are needed to confirm our findings and improve the robustness of the conclusions. Secondly, the definition of seizure cessation across included trials. While most studies relied on clinical observation to determine seizure cessation, a few incorporated EEG confirmation. The subjective nature of clinical assessment may have introduced variability in outcome reporting. Future trials should consider implementing standardized seizure cessation criteria, incorporating both clinical and EEG confirmation, to improve consistency and reliability. The studies included in this analysis also varied in terms of the drug dosage and study methods, mainly for levetiracetam (the dosage varied from 20 to 40 mg/kg). Infusion times also differed between studies. Thirdly, Some of the included studies did not report adverse events, which may have introduced selection bias in our safety analysis. The lack of standardized adverse event reporting could lead to an underestimation of the true incidence of side effects. This limitation highlights the need for future trials to adopt uniform safety reporting criteria to ensure a more accurate assessment of drug-related adverse events. Finally, the studies included in the analysis were predominantly conducted in India and Pakistan, which may introduce regional differences in drug availability, healthcare infrastructure, and prescribing practices. Additionally, population-specific genetic factors may influence treatment response, limiting the generalizability of our findings to other regions. Future research should aim to incorporate data from a broader range of geographic locations to enhance external validity.

## Conclusion

In conclusion, our study showed that levetiracetam has comparable efficacy outcomes and is associated with fewer adverse events and seizure recurrence rates compared with phenytoin/fosphenytoin when used in the treatment of children with CSE.

## Data Availability

The original contributions presented in the study are included in the article/supplementary material, further inquiries can be directed to the corresponding author.
